# Psychopathology in mothers of children with pathogenic Copy Number Variants

**DOI:** 10.1136/jmg-2022-108752

**Published:** 2022-11-29

**Authors:** Maria Niarchou, Adam C. Cunningham, Samuel J. R. A. Chawner, Hayley Moulding, Matthew Sopp, IMAGINE-ID, Jeremy Hall, Michael J. Owen, Marianne B.M. van den Bree

**Affiliations:** 1 Vanderbilt Genetics Institute, Department of Medicine, Vanderbilt University Medical Center, Nashville, Tennessee, USA; 2 MRC Centre for Neuropsychiatric Genetics and Genomics, Division of Psychological Medicine and Clinical Neurosciences, School of Medicine, Cardiff University, Cardiff, UK; 3 Centre for Human Development Science, School of Psychology, Cardiff University, Cardiff, UK; 4 Biotechnology and Biological Sciences Research Council, Swindon, UK; 5 Psychology Department, University of Southampton, Southampton, UK

**Keywords:** mental disorders, pediatrics, psychiatry

## Abstract

**Background:**

Caring for children with pathogenic neurodevelopmental Copy Number Variants (CNVs) (ie, deletions and duplications of genetic material) can place a considerable burden on parents and their quality of life. Our study is the first to examine the frequency of psychiatric diagnoses in mothers of children with CNVs compared with the frequency of psychiatric problems in age-matched mothers from a large community study.

**Methods:**

Case–control study. 268 mothers of children with a CNV diagnosed in a medical genetics clinic and 2680 age-matched mothers taking part in the Avon Longitudinal Study of Parents and Children study.

**Results:**

Mothers of children with CNVs reported higher frequency of depression, anorexia, bulimia, alcohol abuse and drug addiction problems compared with the age-matched mothers from the community sample. Focusing on psychiatric problems arising immediately after the birth of the index child, we found that the levels of depression symptoms were similar between the two groups (48% in mothers of children with CNVs vs 44% in mothers of the community sample, p=0.43), but mothers of children with CNVs had higher frequency of anxiety symptoms (55%) compared with mothers from the community sample (30%, p=0.03).

**Conclusion:**

Our study highlights the need for healthcare providers to devise treatment plans that not only focus on meeting the child’s needs but also assess and, if needed, address the mental health needs of the parent.

**WHAT IS ALREADY KNOWN ON THIS TOPIC**

- Caring for children with complex presentations of neurodevelopmental and physical health problems can be taxing for the mothers. High levels of emotional distress have previously been reported for mothers of children with developmental disabilities. An increasing number of children are now diagnosed with submicroscopic pathogenic CNVs, which increase risk of a range of mental health conditions. There is, however, a paucity of research evaluating the mental health of the mothers of these children.

**WHAT THIS STUDY ADDS**

- Our study is the first to compare the frequencies of mental health symptoms in mothers of children with pathogenic CNVs and age-matched mothers from a large community study. We find that the mothers of children with pathogenic CNVs report more psychiatric problems.

**HOW THIS STUDY MIGHT AFFECT RESEARCH, PRACTICE OR POLICY**

- Our findings highlight the need for the monitoring of the mental health problems of these parents as well as, if indicated, the provision of psychiatric support.

## Introduction

Recent advances in genetic technologies have led to the detection of submicroscopic deletions or duplications of chromosomal regions, otherwise known as CNVs. Certain relatively rare CNVs have been shown to increase the risk of a number of neurodevelopmental and psychiatric conditions, including attention deficit hyperactivity disorder, autism spectrum disorder and anxiety disorder,^1 2^ as well as other health problems.^3^

The multitude of physical and mental health comorbidities associated with pathogenic CNVs in children can place a considerable burden on parental caregiving.^4^ For example, there is evidence that caring for a child with developmental disorder can be taxing on the parent in terms of their emotional (eg, see Mori *et al* 5) and physical well-being (eg, see Laurvick *et al* 6). Research has indicated that mothers with a child with a developmental disability were more likely to experience difficulties coping with stress^7^ and reported worse emotional well-being relative to women from the general population.^8^ Moreover, mothers with these problems were more likely to have children who had more behavioural problems (eg, see Totsika *et al* 9).

Taking into account the ‘diagnostic odyssey’ that parents of children with CNVs often have to go through,^10^ which in turn can impact their quality of life,^11^ as well as the burden of caregiving on the parents placed by the multitude of physical and health comorbidities associated with pathogenic CNVs, there is paucity of research to examine the frequency of psychiatric problems in the parents of children with CNVs. Previous research has indicated high emotional distress in parents of children with CNV.^4^ However, our study is the first to examine the frequency of psychiatric symptoms in mothers of children with CNVs in comparison with age-matched mothers from a large community study. We hypothesised that mothers of children with CNVs have more psychiatric symptoms compared with mothers from the general population. Finally, studies of adults with pathogenic CNVs are indicating they are at a higher risk of psychiatric problems, including internalising disorder.^12 13^ We therefore hypothesised that mothers of children with CNVs who are also carriers of CNVs themselves, would experience more depression and anxiety, compared with mothers without these CNVs.

## Methods

### Samples

#### Mothers of children with CNV

Recruitment of children with CNV was performed through 14 genetics clinics across the UK and the charities Max Appeal, Unique and the 22Crew, as well as social media and word of mouth.

Children’s CNV genotypes were established from medical records as well as in-house genotyping at the Cardiff University MRC Centre for Neuropsychiatric Genetics and Genomics using microarray analysis. Considering our overarching goal of examining the psychopathology of mothers of children with CNVs, we considered it appropriate to also include all children who took part in face-to-face assessments in these studies, meaning that a variety of CNVs were represented. Variants were included if they were (1) pathogenic or likely pathogenic variants according to the American College of Medical Genetics and Genomics guidelines^14^ and/or (2) associated with neurodevelopmental outcomes.^15^

A total of 272 mothers with children with a CNV at the time of assessment (mean age 41.2 years, SD=9.8) and when their child was born (mean age 30.3 years, SD=7.0) were recruited from the Experiences of People with Copy Number Variants (ECHO) study (see https://www.cardiff.ac.uk/mrc-centre-neuropsychiatric-genetics-genomics/research/themes/developmental-psychiatry/echo-study-cnv-research) and the IMAGINE-ID study (http://www.imagine-id.org/). For a list of the children’s CNVs, see online supplemental table 1).

10.1136/jmg-2022-108752.supp1 Supplementary data

For 33% of our sample (n=89) we also had information from the parents on whether the CNV was de novo or was inherited. Ten per cent of the mothers (n=9/89) were CNV carriers.

#### Mothers of children from a community sample

A total of 2680 mothers, matched for their age and the age of their child to the mothers of children with the CNV sample, were recruited from the Avon Longitudinal Study of Parents and Children (ALSPAC). ALSPAC (http://www.bristol.ac.uk/alspac/) is an ongoing population-based cohort which started with inviting pregnant women resident in Avon in the southwest of England, with expected dates of delivery from 1 April 1991 to 31 December 1992, to take part in the study. The initial number of pregnancies enrolled was 14 541 (for these, at least one questionnaire has been returned or a ‘Children in Focus’ clinic had been attended by 19 July 1999). These initial pregnancies resulted in a total of 14 676 fetuses, leading to 14 062 live births with 13 988 children who were alive at 1 year of age.^16 17^

### Measures

#### Mothers of children with CNV

Psychopathology in the mothers of children with CNVs was assessed using the Parental Psychopathology section of the parent version of the Child and Adolescent Psychiatric Assessment (CAPA).^18^ The CAPA is a semistructured interview that generates categorical diagnoses as well as symptom counts of childhood psychopathology. The parental psychopathology section includes questions about a parent’s own psychopathology. Stem questions enquire about whether the parent has ever had depression, anxiety, panic, eating disorder, obsessive compulsive disorder (OCD), psychosis, or problems with alcohol or drugs. If the parent answered ‘yes’ to any of these questions, subsequent questions were asked enquiring about the onset and the severity of the problem (ie, whether the subject sought treatment, received medication or was hospitalised). Information regarding onset was only coded by the rater if the parent could report an exact year of onset; therefore, age at onset data were missing in some instances. For more details, see https://devepi.duhs.duke.edu/measures/the-child-and-adolescent-psychiatric-assessment -capa/.

#### Mothers from community sample

Various questionnaires were administered to mothers at different time points of their child’s development. To enable comparisons with the ECHO study, we selected questions included in the questionnaires that (1) were phrased either identically or as close as possible to the questions asked in the CNV study and 2) were administered when the children and the mothers in the ALSPAC study were within a similar age range to the children and the mothers of the CNV study, in order to facilitate matching (see the Data Analysis section). For these reasons, we selected the mental health-related questions that were included in the ‘Mother and family’ questionnaire (Section A–standard ALSPAC health related questions; link to the questionnaire: http://www.bristol.ac.uk/media-library/sites/alspac/migrated/documents/ques-m13-mother-and-family.pdf) that was administered to mothers when their child was 8 years old, as the mean age of the mothers and the children was similar to the mean age of the mothers and children in the ECHO study. An example of the format of the questions is as follows: Have you ever had any of the following problems: bulimia (‘yes, had it recently’ (‘in past year’)/‘yes, in past, not recently’/‘no never’). The problems we included in our study were severe depression, bulimia, anorexia, alcohol abuse, drug addiction and anxiety. We considered as a ‘yes’, either ‘yes recently’ or ‘yes’ in the past’ answers. Taking into account that in the CNV study the questions about depression included treatment and hospitalisation, we compared this question with the question ‘Have you ever had severe depression’, which was included in the aforementioned questionnaire that was administered to mothers when their child was 11 years old.

### Data analysis

We ran descriptive statistics using R V.3.6.0. To compare the frequency of psychiatric problems between the two samples, we matched the mothers based on their current age using the R ‘MatchIt’ package, with a proportion of 1 (genetic sample) to 10 (community sample). We then compared the frequencies of each group using χ^2^ tests.

## Results

### Psychiatric problems in mothers of children with CNV

The sociodemographic characteristics of the mothers of children with CNV are described in table 1. Depression was the most common psychiatric problem, affecting 54% (n=146) of the sample (table 2). Out of those, the majority sought treatment (84%, 121/144), and received medication (74%, 108/145), while 1% (13/132) was hospitalised. Of the 146 mothers reporting depressive symptoms, 88 indicated the onset, and of those, 43/88 (49%) reported their depressive symptoms started after the birth of their child with a CNV. Anxiety symptoms were reported by 31% (n=85) of the sample. Similar to depression, the majority of individuals sought treatment (73%, 61/83), and 55% (46/84) received medication for their anxiety, while 4% (3/84) were hospitalised. Of the 85 mothers who reported anxiety symptoms, 49 indicated the onset and of those, 55% (27/49) reported their anxiety started after the birth of their child. Other psychiatric symptoms reported included panics (29%), phobias (21%), anorexia and bulimia (12%), other problems (ie, psychotic or OCD-related disorders) (13%) and alcohol/drug problems (6%). We found no evidence that the frequency of depressive symptoms (χ^2^=0.77, p=0.38) or anxiety (χ^2^=0.01, p=0.92) was higher in mothers who also carried a CNV themselves (n=9), compared with mothers who did not (n=80) (table 3).

**Table 1:** Sociodemographic characteristics of mothers of children with CNVs

Family ethnic background (%)	
European	81
Mixed	8
Unknown	11
Highest maternal educational qualification (%)	
Low	7
Middle	43
High	31
Unknown	19
Family income (%)	
<£9999	6
£10 000–£19 999	18
£20 000–£39 000	31
£40 000–£59 000	14
£60 000+	17
Unknown	14

**Table 2:** Frequency of psychiatric problems in mothers of children with a CNV (n=272)

Psychiatric problems		After birth of child
	**N (%**)	**N (%**)
Depression	146 (54)	43 (49)
Sought treatment	121 (84)	
Received medication	108 (74)	
Hospitalised	13 (1)	
Anxiety	85 (31)	27 (55)
Sought treatment	61 (73)	
Received medication	46 (55)	
Hospitalised	3 (4)	
Panics	77 (29)	19 (49)
Sought treatment	39 (48)	
Received medication	31 (39)	
Hospitalised	2 (3)	
Phobias	56 (21)	5 (16)
Sought treatment	21 (34)	
Received medication	12 (19)	
Hospitalised	0	
Anorexia/bulimia	31 (12)	5 (28)
Sought treatment	15 (48)	
Received medication	3 (10)	
Hospitalised	2 (7)	
Other problems	34 (13)	7 (37)
Sought treatment	20 (53)	
Received medication	7 (18)	
Hospitalised	2 (5)	
Drink/drug problems	15 (6)	1 (10)
Sought treatment	3 (20)	
Received medication	1 (7)	
Hospitalised	2 (13)	

N varies between instances, as not all participants responded to all items. The percentage of the ‘after the birth of child’ variable reflects the percentage out of those with the disorder.

**Table 3:** Comparisons of psychiatric symptoms’ frequencies between mothers of children with a CNV and mothers from the community sample, as well as before and after the birth of the child

	Mothers of children with a CNV	Mothers from the community sample	χ^2^	P value
	Nsample/Ntotal (%)	Nsample/Ntotal (%)		
	N=268	N=2680		
Ever depression	142/268 (53)	319/2680 (13)	311.69	<0.001
Sought treatment	120/142 (45)		207.74	<0.001
Medication	105/142 (39)		147.2	<0.001
	**N=246**	**N=2460**		
Anorexia	21/245 (9)	59/2460 (2)	29.4	<0.001
Bulimia	17/245 (7)	61/2460 (3)	15.7	<0.001
	**N=242**	**N=2420**		
Alcohol abuse	10/242 (4)	21/2420 (1)	20.4	<0.001
	**N=244**	**N=2440**		
Drug addiction	7/244 (3)	11/2440 (0)	19.5	<0.001
After the birth of the child*				
	**N=88**	**N=176**		
Depression	43/88 (48)	77/176 (44)	0.62	0.43
	**N=84**	**N=840**		
Anxiety	27/57 (55)	184/656 (30)	4.5	0.03
	**N=9**	**N=80**		
Depression	3 (33)	39/80 (44)	0.77	0.38
Anxiety	2 (22)	19/80 (24)	0.01	0.92

Individuals were matched for their age at the time their child was born. Five mothers of children with CNV included in previous tables did not have data on age; therefore, they were not included in this analysis. There was no question available in ALSPAC that we could directly map to the Experiences of People with Copy Number Variants study related to ‘ever had anxiety disorders’, but there was a question, ‘have you had anxiety since the birth of your child’ which is included in the ‘after the birth of the child’ analyses.

*In the ‘After the birth of the child’ table, we selected mothers who had a psychiatric diagnosis and also data on whether the diagnosis was before or after the birth of their child. Out of 268 mothers of children with CNV, 88 mothers had complete information on whether their psychiatric diagnosis was before or after the birth of their child. They were matched in a 1:2 ratio with mothers from the community sample, because the total of the mothers from the community sample with data on depression and whether it was before or after the birth of their child was n=863.

### Comparisons between mothers of children with CNV and community mothers

We did not have data on age for five mothers of children with CNV; therefore, our comparison of the frequency of depressive symptoms was based on 268 mothers of children with CNV and 2680 age-matched community mothers. Because the question in the community sample did not correspond exactly to the question completed by the mothers of children with CNVs (see the Methods section), we compared the frequency of depressive symptoms in this group, with all three levels of the depression-related question in the mothers of children with CNV sample (table 3). Mothers of children with CNV had higher frequency of all the three levels of depressive symptoms compared with mothers from the community (p<0.001). Furthermore, mothers of children with CNV reported higher frequency of anorexia, bulimia, alcohol abuse and drug addiction symptoms, compared with mothers from the community sample.

The frequency of depressive symptoms arising post the birth of the index child was similar between mothers of children with CNV (48%) and mothers from the community sample (44%, p=0.43). There was, however, evidence of a difference in the frequency of anxiety symptoms arising post the birth of the index child (55% mothers of children with CNV vs 30% mothers from the community sample, p=0.03).

## Discussion

Our study is the first to compare the frequency of psychiatric problems between mothers of children with CNVs and an aged-matched sample of mothers from a community study. The findings supported our hypothesis. Psychiatric problems, including depression, eating disorders and substance abuse/addiction, were more frequently reported by the mothers of children with CNVs, compared with mothers from the community sample. Studies in the general population (eg, see Goodman and Gotlib^19^) as well as on 22q11.2 deletion syndrome (22q11.2DS)^20^ have indicated associations between parental and child psychopathology. However, the nature and direction of these associations is not yet clear. For example, it is possible that maternal psychiatric problems may increase the risk of psychiatric problems in the child via shared genetic background or via the family environment. Studies on 2q11.2DS, a pathogenic CNV associated with a 25-fold increased risk for schizophrenia, have indicated that environmental factors are related to the child’s mental health difficulties (eg, see Shashi *et al* and Allen *et al* 21 22). For example, a study by Allen *et al* 22 found that better parental organisation was associated with fewer difficulties in children with 22q11.2DS. Maternal psychiatric problems may contribute to a less structured family environment that could in turn increase risk of psychiatric problems in the child. On the other hand, it is also possible that the increased physical and mental health problems associated with the CNV in the child may increase the risk of depression and anxiety in the parent due to the high demands on parental caregiving. Longitudinal studies in families of children with developmental disabilities have indicated bidirectional relationships between parenting stress and child behaviour problems.^23^ Futures studies are needed to examine the relationship between parental psychopathology, family environment and child psychopathology in families of children with CNVs, cross-sectionally as well as longitudinally.

We did not find differences in the frequency of depressive and anxiety symptoms between mothers who were CNV carriers themselves compared with mothers who were not. This result is likely due to low power given the small sample sizes. Future studies are needed to better understand how maternal psychopathology may be related to child psychopathology in the context of pathogenic CNVs.

Anxiety symptoms were more frequently reported by mothers of children with CNV after the birth of their child with a CNV, compared with mothers from the community sample.

Our study highlights the need for the monitoring and early intervention for mental health symptoms as well as the effective management of established conditions, not only in children with neurodevelopmental risk CNVs but also their mothers. Indeed, our findings are in line with studies providing evidence that bringing up a child with a neurodevelopmental disorder can be taxing on the parent in terms of their emotional (eg, see Mori *et al* 5) and physical well-being (eg, see Laurvick *et al* 6). Research has identified that mothers with a child with a developmental disability were more likely to experience difficulties with coping with stress,^7^ as well as worse emotional well-being than women from the general population.^8 9^

Our study has some limitations. There are ascertainment differences across the studies (mothers of children with CNV were recently recruited because of their child’s genetic diagnosis, whereas ALSPAC mothers were originally recruited because they were pregnant and invited to take part in a birth cohort study). Also, the fact that children with CNVs were recruited from genetics clinics might indicate that they were enriched for more severe cases and parental distress, and as such, the rates of psychopathology may not be representative of the general population. Furthermore, there were also differences in the psychiatric assessment measures used in the two groups, and it was not possible to validate the questions asked in the community sample in the CNV sample or vice versa. It is therefore possible that we are over-reporting or under-reporting the differences between the two samples. Additionally, age of onset was not available for approximately 40% mothers of children with CNVs who reported depressive or anxiety symptoms. We do not know whether and how this relatively high non-response rate may have affected findings. Another limitation is that we did not examine whether and how the children’s psychopathology may influence their mothers. Future studies are needed to replicate and expand our results. Finally, given the age range of our samples, new incidences of depression are likely to occur. Thus, our study is likely to underestimate the overall frequency of depression in both samples.

In conclusion, we found higher frequencies of depressive symptoms and a number of other psychiatric problems in the mothers of children with CNVs compared with age-matched mothers from the community sample. We also found that the frequency of anxiety symptoms was higher in mothers of children with CNV after the birth of their child with a CNV, compared with mothers from the community sample. Our findings highlight the need for the monitoring as well as, where indicated, effective management of the mental health problems not only of the children with pathogenic CNVs but also their parents.

## Data Availability

No data are available. Due to data sharing restrictions, the data will not be publicly available; however, all scripts used in the study are available upon request.
