# Supplementary material for: Psychopathology in mothers of children with pathogenic Copy Number Variants
Source: J Med Genet. 2022 Nov 29;60(7):706–11. doi: 10.1136/jmg-2022-108752 (PMC10359584; doi:10.1136/jmg-2022-108752)
Supplement: Supplementary data [file jmg-2022-108752supp001.pdf]

Supplementary table 1. CNVs of participants' children

**CNV**	**N**
**11q14.1 deletion**	**2**
**13q12.2 duplication**	**1**
**15q11.2 deletion**	**22**
**15q11.2 duplication**	**1**
**15q13.2 duplication**	**2**
**15q13.3 deletion**	**11**
**15q13.3 duplication**	**10**
**16p11.2 deletion (and distal deletion)**	**37**
**16p11.2 duplication**	**12**
**16p12.2 deletion**	**2**
**16p13.11 deletion**	**4**
**16p13.11 duplication**	**2**
**1q21.1 deletion**	**13**
**1q21.1 duplication**	**17**
**22q11.2 deletion**	**80**
**22q11.2 duplication**	**19**
**22q12.3 duplication**	**1**
**22q13.3 deletion**	**2**
**2p16.3 deletion**	**1**
**3q29 deletion**	**4**
**4q28.3**	**1**
**7p22.1 deletion**	**1**
**7q11.23 duplication**	**1**
**8q12.3 deletion**	**1**
**9q34.3 deletion**	**10**
**17q23 duplication**	**1**
**SHANK3 deletion**	**1**

**Xp21.1**	**1**
**XXXY**	**1**
**NRXN1**	**12**
We note here the primary CNV for which the family was recruited. 12% of our sample had additional CNVs.	
